# Elongation Patterns of the Posterior Cruciate Ligament after Total Knee Arthroplasty

**DOI:** 10.3390/jcm9072078

**Published:** 2020-07-02

**Authors:** Seyyed Hamed Hosseini Nasab, Colin Smith, Pascal Schütz, Barbara Postolka, Stephen Ferguson, William R. Taylor, Renate List

**Affiliations:** 1 Institute for Biomechanics, ETH Zurich, 8093 Zurich, Switzerland; seyyed.hosseini@hest.ethz.ch (S.H.H.N.); colin.smith@hest.ethz.ch (C.S.); ps@ethz.ch (P.S.); barbara.postolka@hest.ethz.ch (B.P.); sferguson@ethz.ch (S.F.); rlist@ethz.ch (R.L.); 2 Human Performance Lab, Schulthess Clinic, 8008 Zurich, Switzerland

**Keywords:** posterior cruciate ligament, knee implant, function, strain, dynamic activity, video-fluoroscopy

## Abstract

This study aimed to understand the ability of fixed-bearing posterior cruciate ligament (PCL)-retaining implants to maintain functionality of the PCL in vivo. To achieve this, elongation of the PCL was examined in six subjects with good clinical and functional outcomes using 3D kinematics reconstructed from video-fluoroscopy, together with multibody modelling of the knee. Here, length-change patterns of the ligament bundles were tracked throughout complete cycles of level walking and stair descent. Throughout both activities, elongation of the anterolateral bundle exhibited a flexion-dependent pattern with more stretching during swing than stance phase (e.g., at 40° flexion, anterolateral bundle experienced 3.9% strain during stance and 9.1% during swing phase of stair descent). The posteromedial bundle remained shorter than its reference length (defined at heel strike of the level gait cycle) during both activities. Compared with loading patterns of the healthy ligament, postoperative elongation patterns indicate a slackening of the ligament at early flexion followed by peak ligament lengths at considerably smaller flexion angles. The reported data provide a novel insight into in vivo PCL function during activities of daily living that has not been captured previously. The findings support previous investigations reporting difficulties in achieving a balanced tension in the retained PCL.

## 1. Introduction

Posterior cruciate retaining total knee arthroplasty (PCR-TKA) has been advocated as a surgical advancement to better restore joint function. The retained posterior cruciate ligament (PCL) is thought to assist femoral rollback with knee flexion, which can consequently increase the moment arm of the knee extensor muscles and increase the knee range of motion (ROM) [1,2,3,4]. However, systematic reviews and meta-analysis of randomised controlled trials indicate no clinically relevant differences between functional and clinical outcomes for PCR-TKA over PCL sacrificing TKA (PCS-TKA) [5,6,7,8,9]. Moreover, several biomechanical studies suggest that femoral rollback, knee kinematics, and PCL loading is largely variable after PCR-TKA [2,4,10,11,12]. Unexpectedly, there are also some articles reporting paradoxical anterior translation of the tibiofemoral contact points with increasing knee flexion after PCR-TKA [13,14]. Here, it has been proposed that abnormal function of the retained PCL may be responsible for variabilities observed in postoperative kinematics [2,15,16].

A comparison of PCL function in healthy vs. PCR-TKA knees reported in the literature indicates that PCR-TKAs may subject the PCL to abnormal loading. Our previous systematic review and meta-analysis of the literature was able to extract the average loading patterns of the natural PCL, and reported that the ligament is increasingly stretched as the knee flexes, before peaking at 90°, after which it shortens [17]. However, a comprehensive review of the literature undertaken here (Table S1, Supplementary Materials) suggests inconclusive findings in post-TKA PCL loading patterns. Both postoperative tightening and loosening of the ligament have been reported [6,11,12,18,19]. Moreover, while some of the reviewed studies reported a peak in postoperative PCL force/strain near 90° knee flexion, other investigations found the postoperative PCL strains to peak at substantially smaller or larger flexion angles (Table S1, Supplementary Materials). This suggests that PCR-TKA can result in abnormal PCL loading, and ultimately may not restore healthy knee function.

Most studies reporting PCL loading after PCR-TKA examined cadaveric specimens using a variety of different assessment techniques (Table S1, Supplementary Materials). As such, inconsistent measurement techniques, implant designs, loading conditions, and outcome measures may partially contribute to the variability observed in reported postoperative PCL loading patterns. Here, in vivo data on post-TKA PCL force/strain can provide a more clinically relevant understanding of the ligament loading, but such investigations have rarely been performed in vivo or during functional activities. Until now, the only assessment of absolute PCL strain in living TKA subjects was reported by Lotke and co-workers [11], where measurements were performed intraoperatively during passive knee flexion from 0° to 120°. Using a Hall Effect Strain Transducer (HEST) attached to the anterolateral (AL) bundle of PCL, they measured largely variable postoperative strain patterns for 10 studied subjects. Maximum strain of the AL bundle ranged from 6% to 18%, which was observed between 45° and 60° of knee flexion. Whether these results indicate real variation in the postoperative PCL load or rather reflect dissimilarities in guidance offered by surgeons is unclear. Thus, a reliable evaluation of post-TKA PCL loading patterns during daily activities is crucial before functionality of the ligament can be fully understood.

Fluoroscopic imaging and image processing now enable the relative kinematics of the tibial and femoral implant components to be measured during restricted movements [20,21,22]. By using the reconstructed 3D kinematics of the joint, tracking of the relative displacements of attachment footprints then allows the elongation patterns of ligaments to be assessed [18,23,24,25]. This technique has similarly been applied to study the function of the intact PCL as well as post-PCR-TKA to show altered postoperative elongation patterns during deep lunge [18]. However, due to the limitations of stationary fluoroscopy systems, PCL function during more functional activities such as level walking and stair descent have not yet been investigated. Recently, the development of the moving fluoroscope at ETH Zürich (Zürich, Switzerland) [26,27] has allowed the knee to be tracked throughout dynamic activities, hence enabling X-ray images of the joint to be captured throughout complete cycles of dynamic activities. Accordingly, the aim of this study was to assess PCL elongation patterns in a group of subjects with PCR-TKA implants during complete cycles of level walking and stair descent.

## 2. Methods

Six subjects (mean age ± SD: 72.8 ± 8.5 years; mean BMI ± SD: 24.3 ± 2.2 kg/m²; 1 female and 5 male) with a unilateral PCR-TKA (PFC Sigma Curved fixed-bearing, DePuy Synthes, Johnson and Johnson, Warsaw, Indiana, USA), each with a neutral mechanical alignment were recruited to take part in this study. Subjects had good postoperative functional and clinical outcomes (mean Knee injury and Osteoarthritis Outcome Score, KOOS ± SD: 91.2 ± 5.7, VAS < 2) and were measured at least 1 year after surgery (mean time ± SD: 4.2 ± 3.5 years). Each subject provided written, informed consent before participation in this study, which was approved by the local ethics commission (EK 2011-N-6).

**Figure 1:**
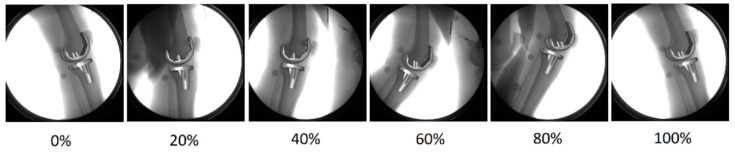
Subject-specific 3D implant geometries were registered to the 2D images of the knee captured by the moving fluoroscope. Exemplary images show a subject’s knee at different time points during a level gait cycle (time as a percentage of the gait cycle is reported below each picture).

A moving fluoroscope [26,27] was used to capture fluoroscopic images of the knee during five complete cycles of each level walking and stair descent. Six degrees of freedom (DoF) implant kinematics were determined using a 2D/3D registration approach [28] to fit the 3D implant geometries to the 2D radiographic images (Figure 1). A detailed description of the implant kinematics has been reported by Schütz and co-workers [29].

**Figure 2:**
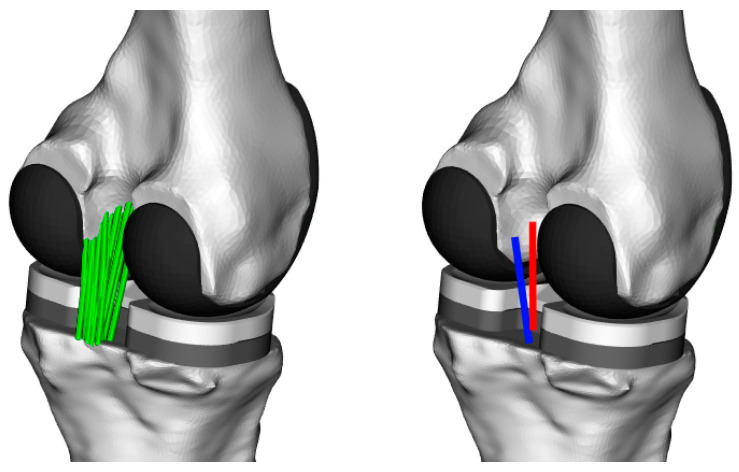
A schematic image of the multibody knee model including two posterior cruciate ligament (PCL) bundles (each represented by 10 fibres) and a 6-degrees-of-freedom (DoF) knee joint (**left**). To summarise bundle function, elongation patterns are reported as the average of the 10 fibres for the anterolateral (AL) (red) and posteromedial (PM) (blue) bundles of the PCL (**right**).

A previously developed multibody knee model [30] was scaled to match the measured subjects’ anthropometry. Subject-specific models were created by scaling tibial and femoral bone geometries and consequently the PCL attachment sites (Figure 2). Within the OpenSim modelling environment [31], skin marker locations captured using an optical system (Vicon, OMG, Oxford, UK) during standing trials were used to scale the generic bones in the superior–inferior direction. The bones were additionally scaled in the mediolateral and anteroposterior directions based on the subject-specific implant dimensions. These anatomical models were then implanted with the 3D CAD model of the subject-specific PFC Sigma implant with mechanical alignment, consistent with the surgery. Finally, a six-DoF tibio-femoral joint was defined between the implant components.

The model represented the anterolateral (AL) and posteromedial (PM) bundles of the PCL using 10 one-dimensional elements (per bundle) connecting their origin and insertion sites. For each activity trial, the fluoroscopically measured 6 DoF tibiofemoral kinematics over time were prescribed to the model tibiofemoral joint. The length of each ligament fibre throughout the entire activity cycle was calculated by the standard analysis tools available within OpenSim, and normalised to their reference length, which was considered to be each fibre’s own length at heel strike of the level walking cycle [32]. For the two studied activities, average and standard deviation (SD) of the elongation patterns were calculated across the 10 fibres representing each bundle, as well as over the trials performed by the six subjects. Finally, the average patterns and their corresponding SDs were presented against the knee flexion angle.

For each ligament bundle, intra-subject variabilities of the ligament elongation patterns were assessed by calculating the SDs between the data obtained for the five trials captured from each subject for every degree of knee flexion. The mean SDs were then calculated by averaging the intra-subject SDs achieved at each degree of knee flexion. Finally, the inter-subject variability was determined at each degree of knee flexion by calculating the average SD across all trials and all subjects. A repeated measures ANOVA based on statistical parametric mapping (SPM [33]) was used to compare the elongation patterns between bundles, phases, and activities.

To assess sensitivity of the ligament elongation patterns to variations in implant kinematics, the 3D poses of the implant components were perturbed around the baseline kinematics captured by the moving fluoroscope during a single gait cycle. Here, the anterio–posterior (AP), medio–lateral (ML) and proximal–distal (PD) translations were varied for each frame within ± 5 mm, while the flexion–extension (FE), varus–valgus (VV), and internal–external (IE) rotations were perturbed within ± 5° (in 1 mm/1° intervals). The perturbed kinematics were then used to drive the subject-specific multibody model and the corresponding elongation patterns were calculated. In addition, change of the maximum ligament elongation due to variation in each implant kinematic parameter was calculated and used to estimate the range of possible errors that might occur from inaccuracies in the employed image-based technique.

## 3. Results

**Figure 3:**
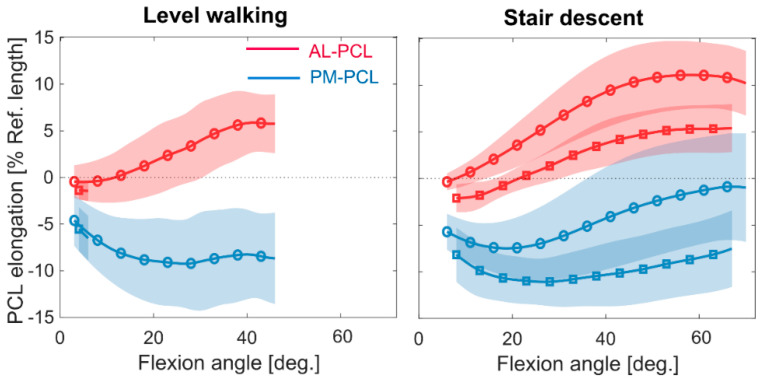
Elongation patterns of the two PCL bundles during stance phase (square markers) and swing phase (circle markers) of level walking (**left**) and stair descent (**right**). Average patterns and standard deviations (shadings) were calculated only over the flexion ranges achieved by all subjects during all the five included cycles.

The intra-subject PCL elongation patterns remained largely consistent between the five repetitions captured in each subject during each of the studied activities (average intra-subject SDs ranging from 0.69% to 1.14%; Figure S1, Supplementary Materials). However, the inter-subject variabilities were relatively large, and were greater at higher flexion angles (average inter-subject SDs ranging from 1.03% to 4.59%; Figure 3, as well as Tables S2 and S3 in Supplementary Materials).

The AL and PM bundles of the PCL exhibited very different elongation patterns throughout both level walking and stair descent (Figure 3). The small overlap between the ranges of flexion covered by all subjects during all trials precluded a complete description of the PCL bundle elongation during the stance phase of level walking. However, between 0° and 6°, both bundles were shorter than their reference lengths at heel strike (mean and standard deviation: 38.8 ± 0.6 mm for AL-PCL and 39.7 ± 1.1 mm for PM-PCL measured at −4.1 ± 3.4° knee flexion). The PCL bundles experienced considerable length changes throughout the range of knee flexion, thus indicating non-isometric behaviour of the ligament during both level walking and stair descent. During level walking, the AL bundle started lengthening at approximately 10° flexion and then showed a gradual lengthening with increasing knee flexion (+5.9 ± 3.2% maximum lengthening at 41°). The PM bundle remained shorter than its reference length throughout the entire swing phase, however, with a shortening until around 27° knee flexion (maximum slackening −9.2 ± 4.8%), with no major length-change thereafter.

**Figure 4:**
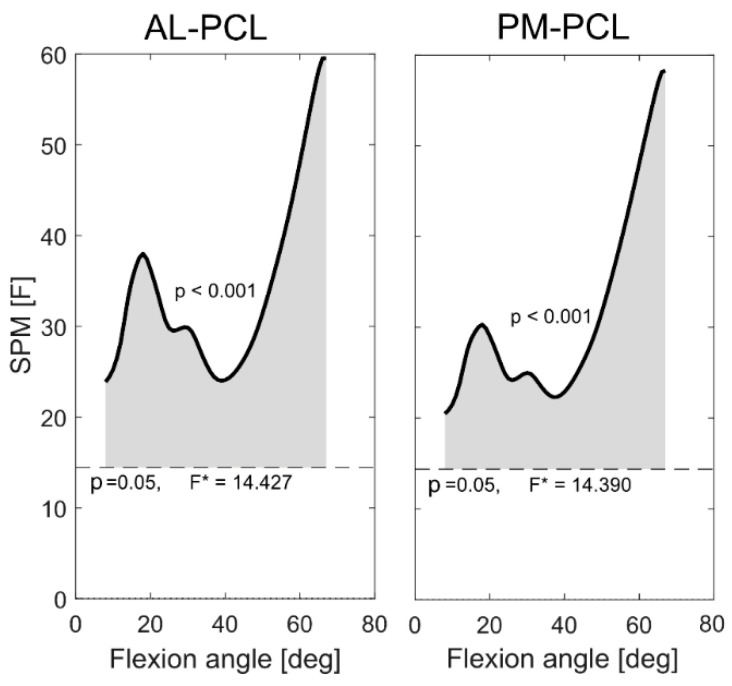
Statistical parametric mapping (SPM) plots show significantly different PCL elongation patterns between the stance and swing phases of stair descent in subjects with posterior cruciate retaining total knee arthroplasty (PCR-TKA).

Throughout the stance and swing phases of stair descent, the flexion dependent ligament elongation patterns exhibited similar trends. Interestingly, however, both bundles were longer during the swing than the stance phase (Figure 3), and this difference was statistically significant over the entire range of knee flexion (Figure 4, *p* < 0.01 for AL and PM bundles). The maximum length-change of the AL bundle during stance phase was 5.4 ± 2.5% observed at 67°, whereas the corresponding value for the swing phase was 11.1 ± 3.6% at 58°. At this 67° angle, the PM bundle was around 8% shorter than its reference length during the stance phase, while at the same flexion angle during the swing phase, it had almost recovered to its reference length.

**Figure 5:**
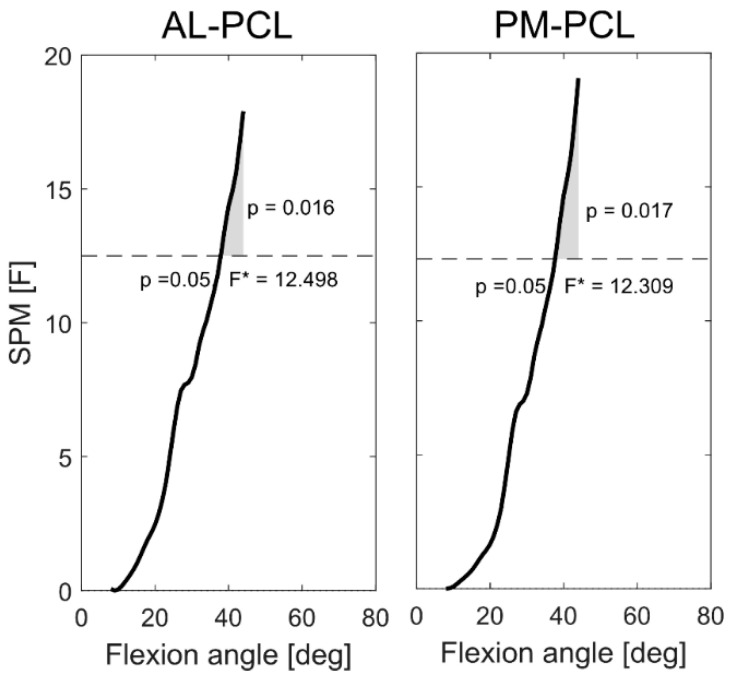
SPM plots show significantly different ligament elongation patterns between level walking and stair descent for flexion angles greater than approximately 40° (SPM tests were performed only over the swing phases of the activities, and within the range of knee flexion covered by all included trials).

At low flexion angles (less than around 40°), the SPM showed no statistically significant differences in the elongation patterns of either PCL bundle between level walking and stair descent (Figure 5). However, both bundles exhibited activity-dependent elongation patterns for flexion angles exceeding approximately 40° (Figure 5, *p* < 0.02 for AL and PM bundles).

**Figure 6:**
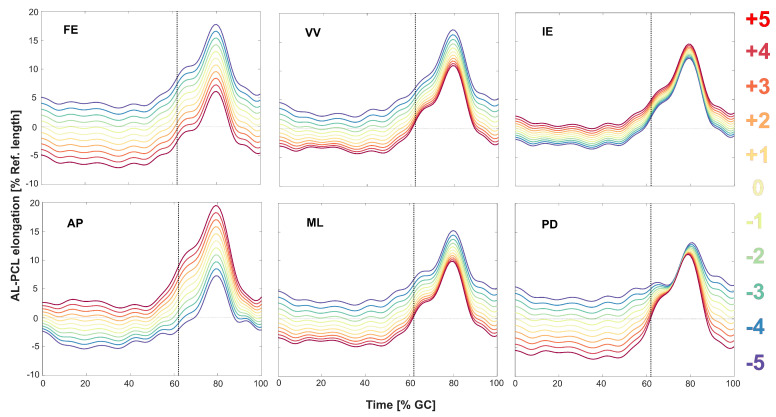
Variation in elongation of the AL-PCL bundle due to perturbing the anterio–posterior (AP), medio–lateral (ML), and proximal–distal (PD) translations as well as the flexion–extension (FE), varus–valgus (VV), and internal–external (IE) rotations of the implant. The baseline kinematics (movement of the tibial component relative to the femoral component) captured from a subject during level walking was perturbed within ±5 mm/±5. The vertical dotted line shows the instant of toe-off.

In general, ligament elongation patterns were partially affected by changes in each of the implant kinematic parameters (Figure 6). Amongst the rotational degrees of freedom (DoFs), variation in flexion–extension (FE) and internal–external rotation (IE) of the implants showed the highest and lowest impacts on the maximum AL-PCL elongation (1.16% and 0.25% change in ligament length, respectively, for 1° variation in FE and IE rotations). The largest impact of variation in the translational DoFs was due to changes in anterior-posterior (AP) displacements of the implant (1.22% change in ligament length per 1 mm shift in AP translation), which was more than twice the influence of perturbing the medio–lateral (ML) translation (0.54% change in ligament length per 1 mm shift in ML). 

## 4. Discussion

Despite the evolution of PCL retaining knee replacement implant designs, it remains unknown whether the natural function of the postoperative ligament is restored, including natural PCL kinematics, throughout activities of daily living. In our study, elongation patterns of the AL and PM bundles of the PCL in six subjects with a fixed bearing PCR-TKA implant were quantified throughout complete cycles of level walking and stair descent. Here, the implant kinematic data, measured using a moving fluoroscope, were used to prescribe the motion of multibody knee models with the PCL bundles represented by a series of one-dimensional elements. Throughout both studied activities, the AL-PCL exhibited more lengthening during the swing than the stance phase. The PM-PCL remained shorter than its reference length (defined as its length at heel strike during level gait) throughout both level walking and stair descent. These data, together with the differences observed between the swing and stance phases, indicate that PCL function in PCR-TKA is critically governed by the loading conditions. As a result, the reported data throughout complete cycles of walking and stair descent, including their loaded and unloaded phases, provide novel insights into in vivo PCL function in activities of daily living. Specifically, our study reveals more elongated PCL bundles during swing than the stance phase, which has not been previously captured, and which suggests that assessment of PCL functionality should be performed throughout complete activity cycles.

Correct intraoperative tensioning of the PCL is consistently emphasised in the literature in order to achieve a successful PCR-TKA [15,34,35]. Ideally, the PCL should restore its normal function to facilitate femoral rollback and restore natural knee kinematics. Regardless of the activity type and loading conditions, stretching of the AL bundle with increasing knee flexion angle, together with no lengthening of the PM bundle until 70°, as observed in our investigation, are generally consistent with the strain patterns reported for the natural PCL [17]. Interestingly, similar to PM-PCL strain patterns in healthy knees [17], the slackest condition of the PM bundle was found at around 20°–30° flexion (Figure 3), and this is probably associated with the posterior translation of the femur relative to the tibia observed at this instant of the gait cycle in this implant [29]. Importantly, however, the slackening of AL-PCL at early flexion (until 10°–20°) post-TKA was not observed in the natural PCL during either passive or active knee flexion [17]. This observation is consistent with previous studies that have also reported a lax PCL at early flexion after PCR-TKA [2,4,11]. PCL slackening at early flexion is plausibly caused by a lack of the anterior cruciate ligament, which is the primary restraint against excessive anterior tibial translation, especially at early knee flexion [36,37]. Moreover, while strain in the natural PCL declines after around 90°, this seems to happen at lower flexion angles (40°–60°) in the studied TKA subjects. Such deviations from natural strain patterns were also reported by Lotke and co-workers during intraoperative passive flexion [11].

Importantly, this study found significant differences in PCL elongation between the swing and stance phases of walking and stair descent. Our results indicate greater lengthening of the AL-PCL during swing than the stance phase. This is once again likely to result from the anterior femoral translation during the swing phase that was reported in the same subjects [29]. These kinematics can be explained by the anterior force of the quadriceps and gastrocnemii on the tibia during stance, and posterior force of the hamstrings during swing [38,39,40]. Unfortunately, beyond the elongation patterns reported during passive activities [17], no relevant data are available that present PCL elongation patterns during loaded dynamic activities in healthy knees, either during the swing or stance phases. As a result, a direct comparison of the influence of the loading conditions and activity type on ligament functionality between healthy and TKA knees is not possible. Here, it is certainly important to consider the time points of highest flexion at the instances where significant differences in PCL function occur as being critical to joint functionality. Although the PCL elongation starts to decline after 40°–60° knee flexion (Figure 3), the PCL remains under tension due to the initial strain existing within the ligament fibres at full extension [34]. On the other hand, however, the PCL plays a key role in guiding femoral rollback, thereby influencing the extensor mechanism, especially at higher flexion angles [1,2,3,4]. Combined with altered joint kinematics, soft tissue structures will therefore be exposed to altered loading conditions to which they were previously not functionally adapted. Therefore, significant differences in ligament elongation patterns between the ligament bundles, tasks or activity phases after 40° are still clinically important and meaningful. As a result, any differences in PCL function at higher flexion angles, even with reduced loading conditions, will be relevant for joint function.

Our comprehensive literature review demonstrated large variability in the reported PCL loading patterns after PCR-TKA (Table S1, Supplementary Materials). A decrease in the PCL tension in PCR-TKA knees at deeper knee flexion angles (80°–100°) was reported in in vitro cadaveric studies that simulated knee bending activities [10,35]. However, such a reduction was not observed in cadaveric knees with PCR-TKA prostheses tested during simulated stair climbing and stair descent [2,4]. Here, it should be noted that previous investigations have been performed on diverse PCR-TKA implant designs, with different methods for the preparation of cadaveric specimens, assessment techniques, loading conditions, and surgical parameters. These factors may explain abnormalities observed in postoperative loading patterns of the PCL in the current study, as well as the large variability in the reported post-TKA PCL strain/force data. Furthermore, they highlight the impact of variations in implant design and surgical techniques on PCL function.

Implant geometry can balance the PCL contributions to femoral roll-back and thereby impact postoperative PCL tension [13,41,42]. To achieve optimal patient outcomes, the implant’s articular geometry should enable the retained PCL to contribute to knee stability, but avoid overtightening of the ligament [42,43]. Lew and Lewis [41] found substantially higher post-operative PCL forces between 60° and 90° of flexion for high- over low-conformity implant designs. In our study, we assessed the PFC Sigma implant, which has substantially lower levels of conformity at larger flexion angles than at full extension. Here, the reduced constraint provided by the implant geometry at higher flexion angles diminishes the contribution of the PCL in providing knee stability, which could explain the decline in the ligament strains observed at these angles (Figure 3). The dependency of PCL elongation and loading patterns on implant conformity may also explain the large variability in reported PCL tension after TKA with various implant designs (Table S1, Supplementary Materials).

The intra- and inter-subject variabilities observed in the elongation patterns obtained in the current study correspond well with variations in the kinematic patterns reported in the same subjects [29]. Here, the very small intra-subject variabilities (Tables S2 and S3 in Supplementary Materials) demonstrate the consistency in elongation patterns in each individual throughout each activity, but also indicate the high levels of reliability of the employed image-based technique. Thus, the relatively large inter-subject variabilities in the PCL elongation patterns (Tables S2 and S3 in Supplementary Materials) can be associated with variations in subject-specific anatomy [44], muscle activation patterns [45], and implantation parameters [46], rather than to any possible inconsistencies in the assessment techniques used. In particular, postoperative alignment of the PCR-TKA components relative to the implanted bones is likely to impact the PCL elongation and loading. Increasing posterior tibial slope (PTS) can prevent over-tensioning of the PCL by shifting the articular contact points posteriorly [47]. Our review of the literature investigating squat activities (Table S1, Supplementary Materials) indicates a decrease in the PCL force in response to increased PTS, and this influence is emphasised at larger knee flexion angles [12,35,48,49,50]. Joint line elevation (JLE) is another surgically controlled parameter that can affect PCL loading after TKA. JLE was found to increase the PCL strain at flexion angles larger than 30° during simulated stair climbing and descent [2,4], while mal-rotation of the tibial component has been shown to play only a minor role in PCL load after PCR-TKA [40]. In addition to adjusting component alignments, surgeons sometimes perform ligament release to balance PCL tension and thereby allow greater knee flexion [51]. However, finite element modelling investigations of such scenarios have suggested a reduction in the PCL force both at mid and deep knee flexion angles due to such partial ligament release [50,52]. Above the general surgical goal of achieving a mechanically aligned TKA, complete details of these important implantation parameters were unfortunately not available for the subjects included in our study. We therefore specifically focussed our study on understanding the changes that occur in PCL elongation between bundles, activities, and loading phases, rather than attempting to determine the absolute strain patterns in the PCL—thereby minimising the uncertainty originating from such unknown surgical parameters. However, any direct comparison between the PCL elongation patterns quantified in this study (Figure 3) with those reported in the literature (Table S1, Supplementary Materials) should still be performed with caution.

There are some other limitations that need to be considered when interpreting the results presented in this study. First, since subject-specific medical images were not available to reconstruct the knee geometry and segment ligament attachment sites, a generic model was scaled to subjects’ anthropometry to represent subject-specific knee models. Moreover, despite delivering highly accurate in-plane kinematics, single-plane fluoroscopy is known to have a limited accuracy in capturing out-of-plane kinematics. The sensitivity analysis performed in this study (Figure 6) indicated that the uncertainties in flexion–extension and anterior-posterior translation indeed impacted the estimated ligament elongation patterns. However, the elongation patterns remained relatively consistent across the trial for each perturbation, hence indicating only small changes to the range and behaviour of bundle elongation. Moreover, high accuracy of the in-plane fluoroscopic kinematics (below 1° and 1 mm error in flexion–extension and anterior-posterior translation [53,54]) ensures small errors in the outcomes. Any potential error in the out-of-plane direction was found to be less critical, regarding the low impact of the medio–lateral translation of the implant on the PCL elongation patterns (Figure 6). Here, a worst-case scenario with 3 mm of error in medio–lateral translation would result in only around 1.6% error in the maximum ligament elongation. In addition to inaccuracies in the fluoroscopic kinematics, image-based assessment of the ligament length does not provide information about the initial (reference) strain within ligament fibres. Thus, the reported elongation patterns should not be interpreted as real in vivo ligament strain patterns that could be measured using strain sensors. However, length-change patterns of virtual bundles connecting origin and insertion sites of the ligaments, as presented in this study, can provide an excellent estimate of the general trend of slackening and lengthening of the ligament [17]. Finally, the elongation patterns of the PCL were quantified only in a small group of subjects with a specific type of PCR-TKA implant, each with a good clinical outcome. As a result, our results may not represent PCL function in subjects with other implant designs or with bad clinical and functional outcomes.

## 5. Conclusions

This study revealed similar trends to the PCL elongation patterns in a group of PCR-TKA subjects to those previously observed in healthy subjects. Here, we additionally show clear differences in the PCL strains under different activities and loading conditions, as well as between the different PCL bundles. However, the differences observed between PCL elongation patterns captured post-TKA and those of the healthy PCL reported in the literature support previous investigations reporting difficulties in achieving healthy tension balance in PCR-TKA.

## Supplementary Materials

The following is available online at https://www.mdpi.com/2077-0383/9/7/2078/s1: Table S1: Summary of the characteristics of the reviewed studies that address post-TKA PCL loads, Table S2: Average intra-subject standard deviations of the ligament elongation patterns (reported in percentage of the ligament reference length), Table S3: Average inter-subject standard deviations of the ligament elongation patterns (reported in percentage of the ligament reference length), Figure S1: Elongation patterns of the anterolateral (AL) and posteromedial (PM) bundles of the PCL during stance phase (left) and swing phase (right) of five level walking trials captured from an exemplary subject. Reference lengths of the ligament bundles were calculated at heel strike.

## References

[1] Hirsch H.S., Lotke P.A., Morrison L.D. (1994). The posterior cruciate ligament in total knee surgery. Save, sacrifice, or substitute?. Clin. Orthop. Relat. Res..

[2] Emodi G.J., Callaghan J.J., Pedersen D.R., Brown T.D. (1999). Posterior Cruciate Ligament Function Following Total Knee Arthroplasty. Iowa Orthop. J..

[3] Andriacchi T.P. (1988). Biomechanics and gait analysis in total knee replacement. Orthop. Rev..

[4] Mahoney O.M., Noble P.C., Rhoads D.D., Alexander J.W., Tullos H.S. (1994). Posterior cruciate function following total knee arthroplasty. A biomechanical study. J. Arthroplast..

[5] Verra W.C., Boom L.G.H.V.D., Jacobs W.C.H., Schoones J., Wymenga A.B., Nelissen R.G.H.H. (2014). Similar outcome after retention or sacrifice of the posterior cruciate ligament in total knee arthroplasty. Acta Orthop..

[6] Li G., Zayontz S., Most E., Otterberg E., Sabbag K., Rubash H.E. (2001). Cruciate-retaining and cruciate-substituting total knee arthroplasty: An in vitro comparison of the kinematics under muscle loads. J. Arthroplast..

[7] Seon J.K., Park S.J., Lee K.B., Yoon T.R., Kozanek M., Song E.K. (2009). Range of Motion in Total Knee Arthroplasty: A Prospective Comparison of High-Flexion and Standard Cruciate-Retaining Designs. J. Bone Jt. Surg. Am. Vol..

[8] Lützner J., Firmbach F.-P., Lützner C., Dexel J., Kirschner S. (2014). Similar stability and range of motion between cruciate-retaining and cruciate-substituting ultracongruent insert total knee arthroplasty. Knee Surg. Sports Traumatol. Arthrosc..

[9] Cho K.-Y., Kim K.-I., Song S.-J., Bae D.-K. (2016). Does Cruciate-Retaining Total Knee Arthroplasty Show Better Quadriceps Recovery than Posterior-Stabilized Total Knee Arthroplasty? - Objective Measurement with a Dynamometer in 102 Knees. Clin. Orthop. Surg..

[10] Wünschel M., Leasure J.M., Dalheimer P., Kraft N., Wülker N., Müller O. (2013). Differences in knee joint kinematics and forces after posterior cruciate retaining and stabilized total knee arthroplasty. Knee.

[11] Lotke P.A., Corces A., Williams J.L., Hirsh H.S. (1993). Strain characteristics of the posterior cruciate ligament after total knee arthroplasty. Am. J. Knee Surg..

[12] Incavo S.J., Johnson C.C., Beynnon B.D., Howe J.G. (1994). Posterior cruciate ligament strain biomechanics in total knee arthroplasty. Clin. Orthop. Relat. Res..

[13] Banks S.A., Hodge W.A. (2004). Implant Design Affects Knee Arthroplasty Kinematics during Stair-stepping. Clin. Orthop. Relat. Res..

[14] Delport H.P., Banks S.A., De Schepper J., Bellemans J. (2006). A kinematic comparison of fixed- and mobile-bearing knee replacements. J. Bone Jt. Surg. Br. Vol..

[15] Cromie M.J., Siston R.A., Giori N.J., Delp S. (2008). Posterior cruciate ligament removal contributes to abnormal knee motion during posterior stabilized total knee arthroplasty. J. Orthop. Res..

[16] Pagnano M.W., Cushner F.D., Scott N.W. (1998). Role of the Posterior Cruciate Ligament in Total Knee Arthroplasty. J. Am. Acad. Orthop. Surg..

[17] Nasab S.H.H., List R., Oberhofer K., Fucentese S.F., Snedeker J.G., Taylor W.R. (2016). Loading Patterns of the Posterior Cruciate Ligament in the Healthy Knee: A Systematic Review. PLoS ONE.

[18] Yue B., Varadarajan K.M., Rubash H.E., Li G. (2012). In Vivo function of posterior cruciate ligament before and after posterior cruciate ligament-retaining total knee arthroplasty. Int. Orthop..

[19] Most E., Li G., Sultan P.G., Park S.E., Rubash H.E. (2005). Kinematic Analysis of Conventional and High-Flexion Cruciate-Retaining Total Knee Arthroplasties. J. Arthroplast..

[20] Schütz P., Taylor W.R., Postolka B., Fucentese S.F., Koch P.P., Freeman M.A., Pinskerova V., List R. (2019). Kinematic Evaluation of the GMK Sphere Implant During Gait Activities: A Dynamic Videofluoroscopy Study. J. Orthop. Res..

[21] Banks S.A., Hodge W. (1996). Accurate measurement of three-dimensional knee replacement kinematics using single-plane fluoroscopy. IEEE Trans. Biomed. Eng..

[22] Grieco T.F., Sharma A., Dessinger G.M., Cates H., Komistek R.D. (2018). In Vivo Kinematic Comparison of a Bicruciate Stabilized Total Knee Arthroplasty and the Normal Knee Using Fluoroscopy. J. Arthroplast..

[23] Hosseini Nasab S.H., Smith C.R., Schütz P., Damm P., Trepczynski A., List R., Taylor W.R. (2020). Length-Change Patterns of the Collateral Ligaments During Functional Activities After Total Knee Arthroplasty. Ann. Biomed. Eng..

[24] DeFrate L.E., Gill T.J., Li G. (2004). In Vivo function of the posterior cruciate ligament during weightbearing knee flexion. Am. J. Sports Med..

[25] Hosseini A., Qi W., Tsai T.-Y., Liu Y., Rubash H., Li G. (2014). In Vivo length change patterns of the medial and lateral collateral ligaments along the flexion path of the knee. Knee Surg. Sports Traumatol. Arthrosc..

[26] Hitz M., Schütz P., Angst M., Taylor W.R., List R. (2018). Influence of the moving fluoroscope on gait patterns. PLoS ONE.

[27] List R., Postolka B., Schütz P., Hitz M., Schwilch P., Gerber H., Ferguson S.J., Taylor W.R. (2017). A moving fluoroscope to capture tibiofemoral kinematics during complete cycles of free level and downhill walking as well as stair descent. PLoS ONE.

[28] Burckhardt K., Szekely G., Notzli H., Hodler J., Gerber C. (2005). Submillimeter measurement of cup migration in clinical standard radiographs. IEEE Trans. Med Imaging.

[29] Schütz P., Postolka B., Gerber H., Ferguson S.J., Taylor W.R., List R. (2019). Knee implant kinematics are task-dependent. J. R. Soc. Interface.

[30] Smith C.R., Vignos M.F., Lenhart R.L., Kaiser J., Thelen D.G. (2016). The Influence of Component Alignment and Ligament Properties on Tibiofemoral Contact Forces in Total Knee Replacement. J. Biomech. Eng..

[31] Delp S., Anderson F.C., Arnold A.S., Loan P., Habib A., John C.T., Guendelman E., Thelen D.G. (2007). OpenSim: Open-Source Software to Create and Analyze Dynamic Simulations of Movement. IEEE Trans. Biomed. Eng..

[32] Liu F., Gadikota H.R., Kozanek M., Hosseini A., Yue B., Gill T.J., Rubash H.E., Li G. (2010). In Vivo length patterns of the medial collateral ligament during the stance phase of gait. Knee Surg. Sports Traumatol. Arthrosc..

[33] Pataky T. (2012). One-dimensional statistical parametric mapping in Python. Comput. Methods Biomech. Biomed. Eng..

[34] Vogrin T.M., Höher J., Årøen A., Woo S.L.-Y., Harner C.D. (2000). Effects of sectioning the posterolateral structures on knee kinematics and in situ forces in the posterior cruciate ligament. Knee Surg. Sports Traumatol. Arthrosc..

[35] Ostermeier S., Schlomach C., Hurschler C., Windhagen H., Stukenborg-Colsman C. (2006). Dynamic in vitro measurement of posterior cruciate ligament load and tibiofemoral stress after TKA in dependence on tibiofemoral slope. Clin. Biomech..

[36] Bell K.M., Rahnemai-Azar A.A.A., Irarrazaval S., Guenther D., Fu F.H., Musahl V., Debski R.R.E. (2017). In Situ force in the anterior cruciate ligament, the lateral collateral ligament, and the anterolateral capsule complex during a simulated pivot shift test. J. Orthop. Res..

[37] Kittl C., El-Daou H., Athwal K.K., Gupte C.M., Weiler A., Williams A., Amis A.A. (2016). The Role of the Anterolateral Structures and the ACL in Controlling Laxity of the Intact and ACL-Deficient Knee: Response. Am. J. Sports Med..

[38] Fleming B.C., Renström P., Ohlen G., Johnson R.J., Peura G.D., Beynnon B.D., Badger G.J. (2001). The gastrocnemius muscle is an antagonist of the anterior cruciate ligament. J. Orthop. Res..

[39] Smith C.R., Brandon S.C.E., Thelen D.G. (2019). Can altered neuromuscular coordination restore soft tissue loading patterns in anterior cruciate ligament and menisci deficient knees during walking?. J. Biomech..

[40] Kuriyama S., Ishikawa M., Furu M., Ito H., Matsuda S. (2014). Malrotated tibial component increases medial collateral ligament tension in total knee arthroplasty. J. Orthop. Res..

[41] Lew W.D., Lewis J.L. (1982). The effect of knee-prosthesis geometry on cruciate ligament mechanics during flexion. J. Bone Jt. Surg. Am. Vol..

[42] Sathasivam S., Walker P. (1999). The conflicting requirements of laxity and conformity in total knee replacement. J. Biomech..

[43] Luger E., Sathasivam S., Walker P.S. (1997). Inherent differences in the laxity and stability between the intact knee and total knee replacements. Knee.

[44] Raheem O., Philpott J., Ryan W., O’Brien M. (2007). Anatomical variations in the anatomy of the posterolateral corner of the knee. Knee Surg. Sports Traumatol. Arthrosc..

[45] Smith J.W., Christensen J.C., Marcus R., LaStayo P.C. (2014). Muscle force and movement variability before and after total knee arthroplasty: A review. World J. Orthop..

[46] Viceconti M., Ascani D., Mazzà C. (2019). Pre-operative prediction of soft tissue balancing in knee arthoplasty part 1: Effect of surgical parameters during level walking. J. Orthop. Res..

[47] Kang K.-T., Koh Y.-G., Jung M.K., Nam J.-H., Son J., Lee Y.H., Kim S.J. (2017). The effects of posterior cruciate ligament deficiency on posterolateral corner structures under gait- and squat-loading conditions. Bone Jt. Res..

[48] Singerman R., Dean J.C., Pagan H.D., Goldberg V.M. (1996). Decreased posterior tibial slope increases strain in the posterior cruciate ligament following total knee arthroplasty. J. Arthroplast..

[49] Takatsu T., Itokazu M., Shimizu K., Brown T.D. (1998). The function of posterior tilt of the tibial component following posterior cruciate ligament-retaining total knee arthroplasty. Bull. Hosp. Jt. Dis..

[50] Zelle J., Heesterbeek P., Malefijt M.D.W., Verdonschot N. (2010). Numerical analysis of variations in posterior cruciate ligament properties and balancing techniques on total knee arthroplasty loading. Med Eng. Phys..

[51] Oshima Y., Fetto J. (2017). Reestablishment of the Posterior Stability After the Posterior Cruciate Ligament-Released Cruciate Retaining Total Knee Arthroplasty. Bull. Hosp. Jt. Dis..

[52] Steinbrück A., Woiczinski M., Weber P., Müller P.E., Jansson V., Schröder C. (2014). Posterior cruciate ligament balancing in total knee arthroplasty: A numerical study with a dynamic force controlled knee model. Biomed. Eng. Online.

[53] Foresti M. (2009). In Vivo measurement of total knee joint replacement kinematics and kinetics during stair descent. Ph.D. Thesis.

[54] Postolka B., List R., Thelen B., Schütz P., Taylor W.R., Zheng G. (2019). Evaluation of an intensity-based algorithm for 2D/3D registration of natural knee videofluoroscopy data. Med. Eng. Phys..

