# Supplementary material for: Elongation Patterns of the Posterior Cruciate Ligament after Total Knee Arthroplasty
Source: J Clin Med. 2020 Jul 2;9(7):2078. doi: 10.3390/jcm9072078 (PMC7408829; doi:10.3390/jcm9072078)
Supplement: Supplementary file 1 [file jcm-09-02078-s001.pdf]

Table 1. Summary of the characteristics of the reviewed studies that address post-TKA PCL loads.

Ref.	Author (year)	Study method	Measurement technique	Subjects characteristics	Studied bundle	Activity	Maximum ligament load
[2]	Emodi (1999)	*In vitro* cadaveric	Direct strain measurement (using a mechanical knee simulator and DVRT)	4 knees (40<age<79) with P.F.C (Johnson and Johnson, Raynham, MA)	AL	An active flexion between 15° and 105° (a simulated stair descent)	8.3 % and 14.7% maximum real bundle strain at 105° flexion for 0mm and 6mm JLE
[12]	Incavo (1994)	*In vitro* cadaveric	Direct strain measurement (using a mechanical knee simulator and DVRT)	8 knees (mean age 57) with Genesis (Smith Nephew Richards, Memphis, TN)	AL	An active flexion between 0° and 70°	2 % and 2.7% maximum bundle strain at 70° flexion for 10°and 0° PTS
[40]	Kuriyama (2014)	modelling	3D musculoskeletal modelling	Fixed bearing (Kyocera, Kyoto, Japan)	PCL	Simulated squat (0°-130°-0°)	1407N maximum force at 120° flexion
[6]	Li (2001)	*In vitro* cadaveric	In situ force measurement (using a 6 DoF robotic knee simulator and UFS)	9 knees (52<age<78) with NexGen (Zimmer, Inc, Warsaw, IN)	PCL	Isometric flexion-extension with 400N quadriceps and 200N hamstrings force	39.2±35.5N maximum in situ PCL force at 90° flexion
[11]	Lotke (1993)	*In vivo*	Direct strain measurement (HEST)	10 subjects (47<age<78) with Miller-Galante (Zimmer, Warsaw, IN) or Press-Fit Condylar (Johnson and Johnson, Raynham, MA)	AL	Intraoperative passive flexion from 0° to 120°	6% to 18% maximum real bundle strain at 45°-60° flexion
[4]	Mahoney (1994)	*In vitro* cadaveric	Direct strain measurement (using a mechanical knee simulator and HEST)	8 knees with Miller-Galante (Zimmer, Warsaw, IN)	AL	An active flexion between 0° and 120° (a simulated stair ascent)	4.5%, 2.4% and 8.2 maximum real bundle strain at 120° flexion for 0mm, -2.5mm and +2.5 mm PTS
						An active flexion between 0° and 110° (a simulated stair descent)	4.1%, 2.6% and 6.6 maximum real bundle strain at 110° flexion for 0mm, -2.5mm and +2.5 mm PTS
[19]	Most (2005)	*In vitro* cadaveric	In situ force measurement (using a 6 DoF robotic knee simulator and UFS)	10 knees (48<age<96) with NexGen CR-Flex (Zimmer, Inc, Warsaw, Ind)	PCL	Isometric flexion-extension with 400N quadriceps and 200N hamstrings force	55.3±29.5N maximum in situ PCL force at 90° flexion

[35]	Ostermeier (2006)	*In vitro* cadaveric	Direct force measurement (using a mechanical knee simulator and bow-shaped force sensor)	12 knees (52<age<78) with Interax I.S.A. (Stryker/Howmedica Limerick, Ireland)	PCL	Isokinetic flexion–extension between 120° and 0° flexion under 31 Nm external moment	29.5±17.1N maximum in situ PCL force at 98° flexion
[48]	Singerman (1996)	*In vitro* cadaveric	Direct strain measurement (using a mechanical knee simulator and DVRT)	7 knees (57<age<76) with Miller-Galante II (Zimmer, Warsaw, IN)	AL	An active flexion between 0° and 120°	2.5%, 3.5% and 5.5% maximum bundle strain at 100° flexion for 10°, 8° and 5° PTS
[52]	Steinbrück (2014)	modelling	3D Finite Element Analysis	Fixed bearing Columbus (Aesculap, Tuttlingen, Germany)	AL	Simulated squat between 0° and 75°	190 N maximum force at 75°
					PM		48 N maximum force at 45°
[49]	Takatsu (1998)	*In vitro* cadaveric	Direct strain measurement (using a mechanical jig and DVRT)	11 knees (69<age<88) with P.F.C (Johnson and Johnson, MA, USA)	AL	An active flexion between 0° and 105°	0.5%, 2.0% and 3.5% maximum bundle strain at 105° flexion for 10°, 5° and 0° PTS

Table S1. Continued.

Ref.	Author (year)	Study method	Measurement technique	Subjects characteristics	Studied bundle	Activity	Maximum ligament load
[10]	Wünschel (2013)	*In vitro* cadaveric	In situ force measurement using a 6 DoF robotic knee simulator and UFS	11 knees (59<age<94) with Genesis II (Smith and Nephew, Memphis NT)	PCL	Simulated squat between 0° and 110°	40 N maximum force at 80°
[18]	Yue (2012)	In vivo	Indirect strain measurement (using static video-fluoroscopy)	11 subjects (51<age<73) with NexGen CR-Flex (Zimmer, Warsaw, IN, USA)	AL	Forward lunge	57% maximum bundle strain at 105°
					PM		42% maximum bundle strain at 105°
[50]	Zelle (2010)	modelling	3D Finite Element Analysis	PFC Sigma CR150 (DePuy International, Leeds, UK)	PCL	Simulated squat between 60° and 150°	600N, 900N, and 1900N maximum force at 150° flexion for 7°, 3° and 0° PTS

Abbreviations: PCL – Posterior Cruciate Ligament; AL – Anterolateral; PM – Posteromedial; DoF – Degree of Freedom; HEST – Hall Effect Strain Transducer; UFS – Universal Force Sensor; DVRT – Differential Variable Reluctance Transducer.

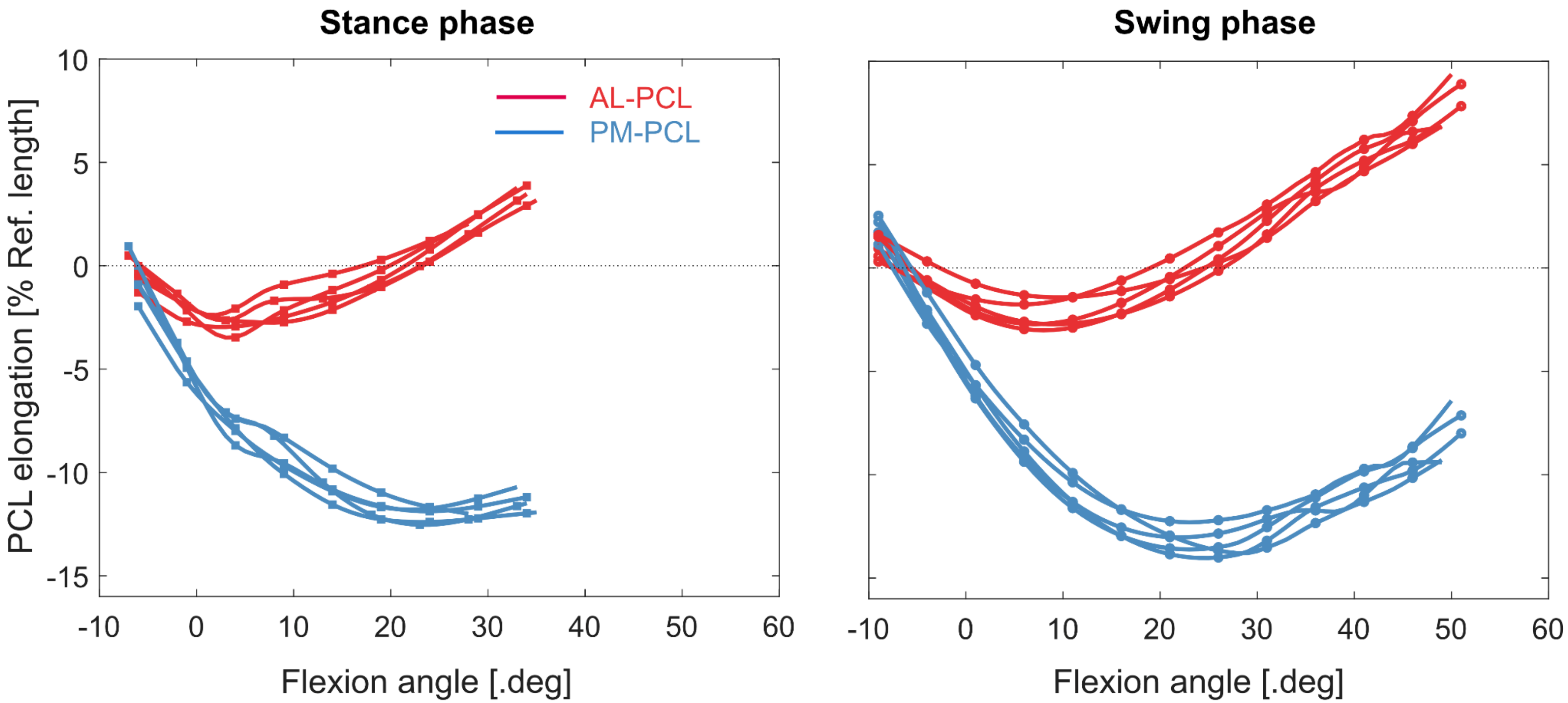

Figure 1. Elongation patterns of the anterolateral (AL) and posteromedial (PM) bundles of the PCL during stance phase (left) and swing phase (right) of five level walking trials captured from an exemplary subject. Reference lengths of the ligament bundles were calculated at heel strike.

Table 2. Average intra-subject standard deviations of the ligament elongation patterns (reported in percentage of the ligament reference length).

	**Level walking**		**Stair descent**	
	Stance	*Swing*	*Stance*	*Swing*
**AL-PCL**	0.69	1.02	0.89	0.75
**PM-PCL**	0.77	1.14	1.05	0.81

Table 3. Average inter-subject standard deviations of the ligament elongation patterns (reported in percentage of the ligament reference length).

	**Level walking**		**Stair descent**	
	Stance	***Swing***	***Stance***	***Swing***
**AL-PCL**	1.03	3.24	2.04	3.02
**PM-PCL**	2.37	4.38	3.23	4.59
